# Supplementary material for: Wet Blue Enzymatic Treatment and Its Effect on Leather Properties and Post-Tanning Processes
Source: Materials (Basel). 2023 Mar 13;16(6):2301. doi: 10.3390/ma16062301 (PMC10051666; doi:10.3390/ma16062301)
Supplement: Supplementary file 1 [file materials-16-02301-s001.zip › materials-2224620-supplementary.pdf]

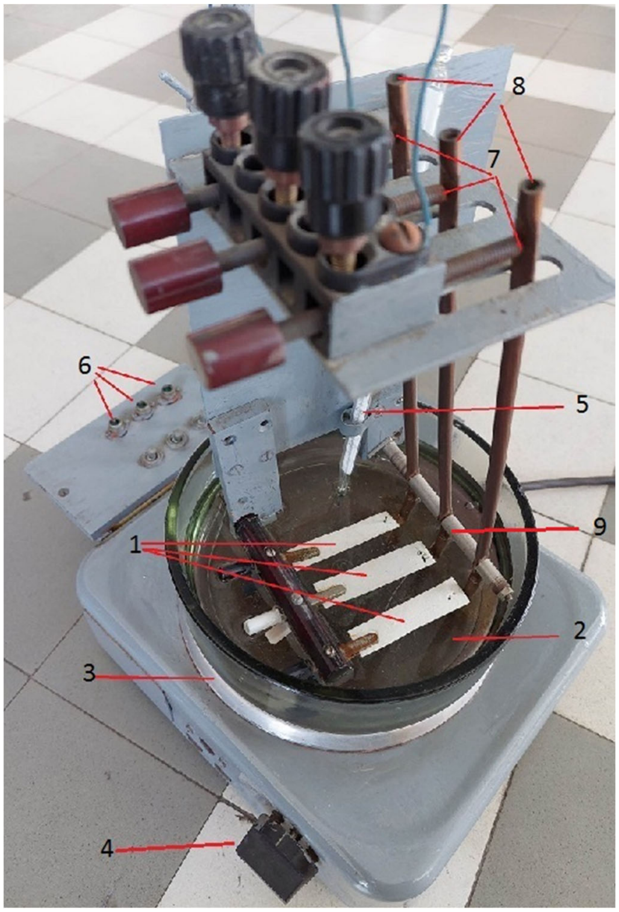

Figure S1. Equipment for determination of leather's shrinkage temperature.

1—leather specimens; 2—vessel with glycerol; 3—heater; 4—temperature increase speed controller; 5—thermometer; 6—light indicators; 7—stationary conductors; 8—levers-conductors; 9—levers' axis-conductor.

Working principle. Three leather specimens (1) (3x50 mm) are fixed in the apparatus and immersed in the vessel with glycerol (2), together with a thermometer (5). Stationary conductors (7) are adjusted so that there is contact between them and the levers and conductors (8). The electric circuit closes, and the electric current flows from the current source through the axis of the levers (9), levers and conductors, and stationary conductors; this is confirmed by illuminated indicators (6). Temperature is increased by a heater (3). The rate of temperature increase must not exceed 5°C per minute.

When the leather shrinkage temperature is reached, the specimens start to shrink rapidly, and contact between the levers and conductors and the stationary conductors disappears; at the same time, the light indicators turn off. At that moment, the temperature shown by the thermometer is recorded as the shrinkage temperature of the samples.
